# Crude extract and isolated bioactive compounds from *Notholirion thomsonianum* (Royale) Stapf as multitargets antidiabetic agents: in-vitro and molecular docking approaches

**DOI:** 10.1186/s12906-021-03443-7

**Published:** 2021-10-27

**Authors:** Mater H. Mahnashi, Yahya S. Alqahtani, Ali O. Alqarni, Bandar A. Alyami, Muhammad Saeed Jan, Muhammad Ayaz, Farhat Ullah, Umer Rashid, Abdul Sadiq

**Affiliations:** 1 Department of Pharmaceutical Chemistry, College of Pharmacy, Najran University, Najran, Saudi Arabia; 2 Department of Pharmacy, University of Swabi, Swabi, KP Pakistan; 3 Department of Pharmacy, Faculty of Biological Sciences, University of Malakand, 18000 Dir (L), KP, Chakdara, Pakistan; 4 Department of Chemistry, COMSATS University Islamabad, Abbottabad Campus, Abbottabad, Pakistan

**Keywords:** *Notholirion thomsonianum*, Bioactive compounds, *α*-Glucosidase, *α*-Amylase, Protein tyrosine phosphatase 1B (PTP1B), Antioxidant

## Abstract

**Background:**

Diabetes mellitus is a common disease effecting the lifestyles of majority world population. In this research work, we have embarked the potential role of crude extracts and isolated compounds of *Notholirion thomsonianum* for the management diabetes mellitus.

**Methods:**

The crude extracts of *N. thomsonianum* were initially evaluated for α-glucosidase, α-amylase and antioxidant activities. The compounds were isolated from the activity based potent solvent fraction. The structures of isolated compounds were confirmed with NMR and MS analyses. The isolated compounds were tested for *α*-glucosidase, *α*-amylase, protein tyrosine phosphatase 1B (PTP1B) and DPPH activities. The molecular docking studies were carried out to find the binding interactions of isolated compounds for *α*-glucosidase, *α*-amylase and PTP1B.

**Results:**

Initially, we screened out crude extracts and subfractions of *N. thomsonianum* against different in-vitro targets. Among all, Nt.EtAc was observed a potent fraction among all giving IC_50_ values of 67, 70, < 0.1, 89 and 16 μg/mL against *α*-glucosidase, *α*-amylase, DPPH, ABTS and H_2_O_2_ respectively. Three compounds (Nt01, Nt02 and Nt03) were isolated from Nt.EtAc of *N. thomsonianum*. The isolated compounds Nt01, Nt02 and Nt03 exhibited IC_50_ values of 58.93, 114.93 and 19.54 μM against α-glucosidase, while 56.25, 96.54 and 24.39 μM against *α*-amylase respectively. Comparatively, the standard acarbose observed IC_50_ values were 10.60 and 12.71 μM against *α*-glucosidase, *α*-amylase respectively. In PTP1B assay, the compounds Nt01, Nt02 and Nt03 demonstrated IC_50_ values of 12.96, 36.22 and 3.57 μM in comparison to the standard ursolic acid (IC_50_ of 3.63 μM). The isolated compounds also gave overwhelming results in DPPH assay. Molecular docking based binding interactions for *α*-glucosidase, *α*-amylase and PTP1B were also encouraging.

**Conclusions:**

In the light of current results, it is obvious that *N. thomsonianum* is potential medicinal plant for the treatment of hyperglycemia. Overall, Nt.EtAc was dominant fraction in all in-vitro activities. Three compounds Nt01, Nt02 and Nt03 were isolated from ethyl acetate fraction. The Nt03 specifically was most potent in all in-vitro assays. The molecular docking studies supported our in-vitro results. It is concluded that *N. thomsonianum* is a rich source of bioactive antidiabetic compounds which can be further extended to in-vivo based experiments.

**Supplementary Information:**

The online version contains supplementary material available at 10.1186/s12906-021-03443-7.

## Background

Diabetes mellitus (DM) is a chronic metabolic disorder characterized by persistent elevated blood glucose levels, which leads to diabetic neuropathy, retinopathy, nephropathy, cardiovascular disorders and other serious complications [1]. DM is among the major health problems, affecting about 171 million people worldwide, of which cases of type 2 diabetes are dominated [2]. Though the drug treatment for type 2 DM has been developed to some extent during the last decade, yet drug resistance is a huge concern that needs to be dealt with efficient approaches [3]. Diminishing of postprandial increased blood sugar level is among the therapeutic options for diabetes [4]. It is a membrane bound intestinal enzyme which catalyzes the release of *α*-glucose from the non-reducing end of the substrate which is subsequently absorbed into the blood [5]. The organic and medicinal chemists are constantly researching for the development of new and effective drug molecules with efficient methods [6, 7]. Various synthetic and natural sources have been explored till date [8, 9]. However, due to the increasing demand of new drugs, it is the need of the day to develop and explore new molecules as potential antidiabetic agents.

Natural products have long been and still are the source of treatment and prevention of different disorders including diabetes [10]. Natural products of vast structural diversity are still best source for the development of such inhibitors, thus motivating to discover biologically active compounds from plants [11]. Currently, the presence of *α*-glucosidase inhibitors (AGIs) like acarbose and voglibose in microorganisms, and nojirimycin and 1-deoxynojirimycin has been reported from plants [12]. Recently, numerous efforts have been made to find out more effective drugs against type 2 diabetes from natural sources to develop physiologic functional food or isolate new and more effective compounds [13]. Several AGIs present as phytoconstituents including alkaloids, glycosides, flavonoids, terpenoids and phenolic compounds have been reported from plant origin [1]. Thus, there is an urgent need to search for novel drugs from several sources including natural products with increased potency and lesser adverse effects than the existing drugs to fight global health problems posed by DM.

Aerobic life utilizes oxygen to metabolize food substrates which are rich in carbon and hydrogen to gain energy necessary for life activities. During the oxidation process, the oxygen molecule itself turns into reduced state and forms intermediates. During physiological metabolism, reactive oxygen species (ROS) are constantly created which are counter-balanced by cellular antioxidant protective mechanisms [13]. These ROS are highly reactive and are able to accommodate or donate electrons to an extensive range of biological molecules. Modern research revealed that ROS may work as the mechanical link for salt sensitive hypertension, high nutrition, fatty diet intake, metabolic syndrome and Type-2 DM in experimental animal models [14]. Several studies confirmed that increased oxidative stress is allied with insulin resistance pathogenesis through inhibition of insulin signals and adipokines dysregulation [15, 16]. Therefore, ROS may also contribute and speed up the development of insulin resistance.

*Notholirion thomsonianum* (Royale) Stapf belong to Liliaceae family. It is traditionally used in the treatment of a variety diseases including tumors, digestive and microbial mediated disorders [17]. We recently have explored the antimicrobial and analgesic potentials of this plant [18, 19]. The plant is not scientifically validated for anti-diabetic activity and very limited exploration has been performed on this plant. The current study was designed to investigate the crude extracts and its bio-guided isolated bioactive compounds as multitargets anti-diabetic agents from *N. thomsonianum*.

## Material and methods

### Chemicals and drugs

All the chemicals and drugs used in this research were purchased from the local vendors of Sigma company.

### Plant collection, extraction and fractionation

*Notholirion thomsonianum* was collected from different regions of Swat KP, Pakistan and was identified by Dr. Nasrullah, Plant Taxonomist at the department of Botany University of Malakand, Pakistan. A sample from the plant was pressed and deposited in herbarium of University of Malakand, Pakistan, under voucher specimen H.UOM.BG.106. As per our previous mentioned procedure, we did extraction and fractionation of the plant’s sample [20].

### In-vitro *α*-glucosidase inhibition

Yeast *α*-Glucosidase inhibitory potentials of the samples from plant extracts and isolated compounds (Nt01 – Nt03) were analyzed using the method reported [9]. Acarbose, a competitive *α*-glucosidase inhibitor, was used as standard drug. In 0.10 M of phosphate buffer having pH of 6.90, the *α*-glucosidase enzyme solution was prepared (0.50 U/ mL). The enzyme solution contains the mentioned amount of *α*-glucosidase and phosphate buffer (0.10 M, 120 μl. In the same phosphate buffer of pH 6.90, 5 mM of *p*-nitrophenyl-*α*-D-glucopyranoside was added as substrate solution. The crude extracts and compounds solution were made in concentrations ranges from 31.25 to 1000 μg/mL. The sample and enzyme solutions were mixed and incubated for 15 min at 37 °C. Finally, 20 μl of the substrate solution was mixed with the enzyme-sample mixture and were again incubated for the same amount of time. At the end, solution of sodium carbonate (80 μl, 0.20 M) was added to complete the reaction. The same solution without the enzyme was used as a blank. The absorbances were recorded at 405 nm. All the experiments were repeated in triplicate. The percent inhibitions and subsequent IC_50_ (in μg/mL and also in μM) were calculated accordingly.

### In-vitro *α*-amylase inhibition

The potential *α*-amylase activities on crude extracts and isolated compounds (Nt01 – Nt03) of *N. thomsonianum* were performed as per the standard procedure [10]. The amylase solution was prepared in phosphate buffer followed by addition of different concentrations of plant extracts and isolated compounds. The solutions of test samples in enzyme solution were allowed to react. The solutions were incubated for a specific temperature and were added starch solution. Afterwards, a solution of dinitrosalicylic acid was added to both of the groups, i.e. sample and control solution groups. The solutions were then allowed to heat up in boiling water for a few min. The absorbances were measured at 656 nm using a microplate reader. The experiments were performed in triplicates and percent inhibitions were calculated as per the reported formula.

### Protein tyrosine phosphatase 1B inhibition

The PTP1B activity was performed only on the isolated compounds Nt01 – Nt03 using the standard procedure [21]. Buffer solution of 3,3-dimethyl glutarate (pH 7) was used in PTP1B assay. The solutions were prepared from para nitrophenol phosphate (1 mM), PTP1B (10 mM) and different strengths of compounds Nt01, Nt02 and Nt03. The solutions were then incubated for 40 min at 27 °C and absorbances were recorded at 405 nm using microplate reader. The experiments were repeated three times and percent inhibitions followed by IC_50_ values were recorded accordingly.

### Antioxidant assays

#### DPPH free radicals scavenging assay

DPPH free radicals scavenging ability of the plant’s samples and isolated compounds (Nt01 – Nt03) were evaluated following previously reported procedures [22]. Plant samples solutions were prepared ranging from 125 to 1000 μg/ mL and were added to 0.004% methanolic solution of DPPH. Subsequent to 30 min incubation, absorbances were measured at 517 nm. The percent inhibitions and IC_50_ values of samples and standard acarbose were calculated as per the protocols.

#### ABTS free radicals scavenging assay

Plant crude samples were also evaluated against ABTS free radicals using standard protocol [23]. In this protocols, 7 mM solution of ABTS and 2.45 mM of potassium per sulfate were mixed and kept in dark at laboratory temperature for around 16 h till the color become dark. Solution of 0.01 M of phosphate buffer having pH 7.40 was mixed with it and absorbance was adjusted at 0.70 at 734 nm. Afterwards, solution of the plant extracts (300 μl) was added to ABTS solution (3 mL) and absorbance was recorded at 734 nm. Mixed the solution for 1 min and reduction in absorbance was recorded. The mixing and reduction were repeated for 6 min. The experiments were performed three times. The percent inhibitions and subsequent IC_50_ values of samples and standard ascorbic acid were calculated as per the standard procedure [24].

#### H_2_O_2_ free radicals scavenging assay

This method was used to determine the antioxidant potentials of crude extracts from *N. thomsonianum*. A solution of H_2_O_2_ (2 mM) was prepared in buffer solution of phosphate (50 mM, pH 7.40). In a test tube, 0.10 mL of crude extract was measured, and the volume was diluted to 0.40 mL by adding the phosphate buffer. Then 0.60 mL solution of H_2_O_2_ was added to it and was mixed properly. The absorbance of the crude sample and standard were recorded on 230 nm. The same procedure was repeated for all the crude samples. The percent inhibitions and IC_50_ values were calculated as per the reported procedure [25].

#### Estimation of IC_50_ values

Samples’ concentrations that inhibited substrate hydrolysis by 50% (IC_50_) were calculated via Microsoft Excel program. In free radicals’ assays including DPPH, ABTS and H_2_O_2_, the IC_50_s were calculated using same method [25].

### Statistical data analysis

All the assays were performed in triplicate and data values were expressed as mean ± S*.*E*.*M. One-way ANOVA followed by Dunnett’s multiple comparison test was applied for the comparison of positive control with the test group using GraphPad prism Software USA [26]. The *P* values less than 0.05 were considered as statistically significant. The values in all in-vitro assays are mentioned as mean ± SEM with *n* = 3. Moreover, values of P less than 0.05 are statistically significant. The P values are compared with the standard drug, such as * = *P* < 0.05, ** = *P* < 0.01 and *** = *P* < 0.001.

### Docking studies

We performed molecular docking simulations using Molecular Operating Environment software (MOE 2016). Three-dimensional structure of *α*-glucosidase was constructed by using homology model technique as per our previously reported procedure. While 3-D structures of *α*-amylase and PTP-1B were obtained from protein data bank with accession codes 4 W93 and 1NNY respectively. Before starting the docking on tested compounds, the protocol for the docking studies was validated by using re-dock method. The measured RMSD values were within reasonable limits (< 2.0 Å). Preparation of downloaded enzymes such as determination of binding sites, energy minimization and 3-D protonation was performed by previously reported methods [14, 27, 28]. Structures of the compounds were built using builder option in MOE software. The built structures were then energy minimized using MMFF94X forcefield and 0.0001 gradient and data base was built. Docking study was carried out using validated parameters (placement / refinement stage and scoring / rescoring functions. Interpretation of docking results was carried out by using MOE and discovery studio visualizer.

## Results

### Extraction and fractionation

The plant materials were extracted with our previous reported method. After the extraction process, the methanolic extract was subjected to rotary evaporator for removal of solvent. The dried methanolic extract (Nt.Cr about 0.30 Kg) of the plant was suspended in 0.50 L of distilled water and was added 0.50 L of *n*-hexane with proper shaking in a separating funnel. The separating funnel was put in a stand and the two layers were given the time to separate. The organic layer (*n*-hexane) was separated, and the same procedure was repeated three times to get enough amount of *n*-hexane fraction. The *n*-hexane layer was subjected to rotary evaporator. The dried *n*-hexane fraction (Nt.Hex) was stored and labelled properly. The same solvent fractionation procedure was performed by using different solvent of increasing the polarity. So, the next fractions we got were of chloroform (Nt.Chf), ethyl acetate (Nt.EtAc), and the final layer obtained was that of aqueous layer (Nt.Aq).

### Isolation of compounds (Nt01 – Nt03)

Based on the overall potency in all in-vitro assays, the ethyl acetate (Nt.EtAc) was selected for the isolation of bioactive compounds. Initially, the Nt.EtAc was loaded on the top of a pre-packed silica gel gravity column. The column was initially eluted with pure *n*-hexane. Afterwards, the polarity of eluent was gradually increased by adding a small amount of polar modifier (ethyl acetate solvent). There was a gradual increase of 2% in polarity of solvent system. At the end, the polarity was increased to 1:1 ratio of *n*-hexane/ethyl acetate. Semi-purified phytochemicals were collected and dried. Three of the semi-purified products were then individually loaded onto small pre-packed silica gel column. Each of the small column was also eluted with *n*-hexane/ethyl acetate. Starting from low polarity and gradually increasing the polarity, we were able to get the purified products (Nt01 – Nt03) as visualized on a TLC plate.

### Structure confirmations of compounds Nt01 – Nt03

**Fig. 1:**
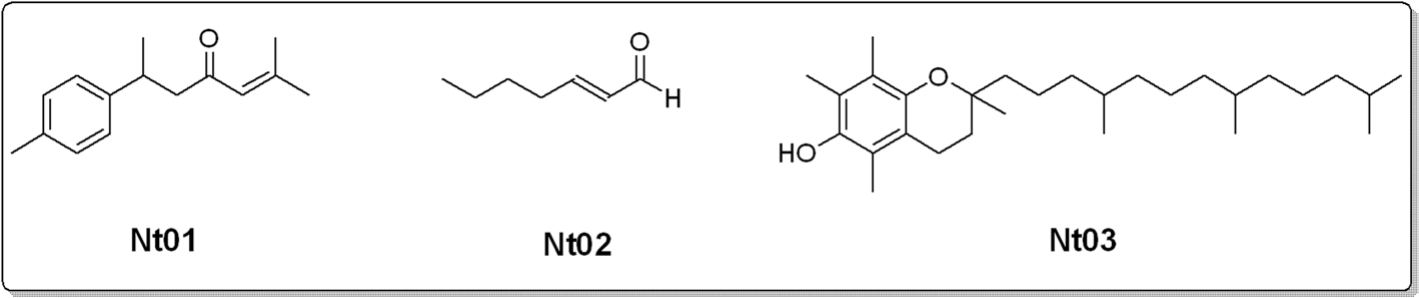
Structures of isolated compounds isolated from ethyl acetate fraction of *Notholirion thomasonianum*

The structures of the isolated compounds were determined with NMR and MS analyses as shown in Fig. 1. The compound Nt01, a para substituted aromatic derivative type compound molecular weight was confirmed as 216 (Figure S1, Supporting information). The compound structure was also confirmed with NMR (Figure S2, Supporting information). The compound Nt01 has four distinct methyl groups in which only one owns a neighbouring H – atom. The methyl group having a neighbouring H – atom gave a doublet of 3 protons at chemical shift of 1.13 with coupling constant value (*J*) of 8.49 Hz. The two methyl groups attached to a double bond gave two singlets at 2.00 and 2.11. The methyl group attached at para position to the benzene ring appeared at chemical shift of 2.27. The single vinylic hydrogen gave a singlet at 5.98. The three aliphatic protons gave a multiplet in the range of 2.93–3.28. The aromatic proton gave the typical splitting pattern of a para substitution by giving two consecutive doublets. The doublets appeared at 7.07 and 7.13 with *J* values of 8.05 and 7.98 Hz respectively. The carbon NMR was also in agreement with the compound’s structure. There were clear signals for aliphatic and aromatic/vinylic carbons. The carbonyl carbon appeared above chemical shift of 200. The MS and ^1^H NMR data of compound Nt02 is shown in Figure S3 and S4 of the Supporting information respectively. The aldehydic protons gave a singlet at 9.64. The vinylic proton at alpha position to the carbonyl gave a doublet at 5.69 with *J* value of 10.66 Hz. The high *J* value of the doublet shows a trans-confirmation. Similarly, the second vinylic H – atom also gave a multiplet at 6.43–6.29. The three methylene (−CH_2_-) appeared in the form multiplets between chemical shift 2.13 and 1.28. The methyl group gave a distinct triplet at 0.97 with *J* value of 7.23 Hz. The MS and ^1^H NMR data of compound Nt03 is shown in Figure S5 and S6 of the Supporting information respectively. The molecular weight was confirmed as 430 and there is a dominant peak at 165 which possibly represent the fragmentation of aromatic moiety out of the molecule. Due to the large number of aliphatic protons in compound Nt03, various splitting patterns arising from methyl groups, methylene and methine units overlap with each other in the chemical shift range of 2.17–0.85. Among these, the nine protons at 2.17 and 2.12 can be attributed to the three methyl groups attached to the benzene ring. Similarly, one of the methylene units attached to the benzene ring gave a triplet at 2.62 with *J* value of 6.83 Hz. The most downfield signal was that from the phenolic H – atom, which gave a broad singlet at 4.21.

### *α*-Glucosidase and *α*-amylase inhibition assays of plant extracts

The *α*-glucosidase and *α*-amylase inhibitory activities of the crude extracts and different solvent extracts of *N. thomsonianum* are summarized in Table 1. In *α*-glucosidase inhibition assay of *N. thomsonianum,* Nt.EtAc was found most active causing 68.57 ± 1.26, 61.89 ± 1.70, 57.77 ± 1.01, 53.61 ± 2.31 and 45.33 ± 0.66% inhibitions at concentrations of 1000, 500, 250, 125 and 62.50 μg/mL respectively attaining IC_50_ of 67 μg/mL. This was followed by Nt. Cr exhibiting 64.78 ± 0.92, 54.11 ± 0.07, 51.55 ± 0.41, 47.55 ± 0.67 and 40.00 ± 0.57% inhibitions at concentrations of 1000, 500, 250, 125 and 62.50 μg/mL respectively with IC_50_ of 185 μg/mL. The other tested samples from *N. thomsonianum* were less effective in an order of Nt. Chf > Nt. Hex > Nt. Aq with IC_50_ values of 394, 435 and 614 μg/mL respectively. In comparison, the observed/calculated IC_50_ value of the standard drug acarbose was 25 μg/mL using the same concentrations.

**Table 1:** *α*-Glucosidase and *α*-amylase inhibitory potentials of *Notholirion thomsonianum*

Samples	Conc (μg/mL)	Alpha glucosidase		Alpha amylase	
		Percent Inhibition (mean ± SEM)	IC_**50**_ (μg/mL)	Percent Inhibition (mean ± SEM)	IC_**50**_ (μg/mL)
Nt.Cr	1000 500 250 125 62.5	64.78 ± 0.92*** 54.11 ± 0.07*** 51.55 ± 0.41*** 47.55 ± 0.67*** 40.00 ± 0.57***	185	62.41 ± 0.24** 57.65 ± 0.01*** 54.62 ± 0.63** 45.32 ± 0.41*** 41.10 ± 0.48***	180
Nt.Hex	1000 500 250 125 62.5	57.44 ± 0.60*** 51.45 ± 0.57*** 44.84 ± 0.39*** 39.13 ± 0.92*** 32.00 ± 1.00***	435	60.52 ± 0.52** 57.52 ± 0.52*** 50.24 ± 0.27** 46.52 ± 0.51*** 38.22 ± 0.82***	240
Nt.Chf	1000 500 250 125 62.5	59.69 ± 1.21*** 53.88 ± 1.04*** 47.28 ± 1.15*** 42.31 ± 0.48*** 35.00 ± 0.00***	394	56.53 ± 1.01*** 51.23 ± 0.86*** 43.52 ± 1.03*** 39.12 ± 0.93*** 36.53 ± 0.52***	450
Nt.EtAc	1000 500 250 125 62.5	68.57 ± 1.26*** 61.89 ± 1.70*** 57.77 ± 1.01*** 53.61 ± 2.31*** 45.33 ± 0.66***	67	71.30 ± 0.83^ns^ 64.42 ± 1.20 ^ns^ 60.52 ± 1.54^ns^ 55.63 ± 1.65^ns^ 48.35 ± 1.08 ^ns^	70
Nt.Aq	1000 500 250 125 62.5	55.77 ± 0.95*** 48.55 ± 1.75*** 39.35 ± 0.21*** 33.75 ± 0.44*** 28.00 ± 0.00***	614	47.53 ± 0.24*** 43.74 ± 1.32*** 37.52 ± 1.54*** 29.32 ± 0.63*** 26.53 ± 0.27***	950
Acarbose	1000 500 250 125 62.5	76.87 ± 0.06 73.94 ± 1.92 67.49 ± 0.22 61.53 ± 0.89 55.50 ± 1.00	6.84	78.63 ± 0.54 71.65 ± 0.71 68.95 ± 1.20 64.52 ± 0.21 57.63 ± 0.59	13

In almost a similar pattern, different extracts from *N. thomsonianum* exhibited the alpha amylase activity as shown in Table 1. Again, the Nt.EtAc fraction was dominant among all the crude fractions. The Nt.EtAc at concentrations of 1000, 500, 250 and 125 μg/mL exhibited percent amylase inhibitions of 71.30, 64.42, 60.52, 55.63 and 48.35 respectively with IC_50_ of 70 μg/mL. In comparison, the standard acarbose gave IC_50_ value of 13 μg/mL. The other fractions like Nt. Cr, Nt. Hex, Nt. Chf and Nt. Aq exhibited IC_50_ values of 180, 240, 450 and 950 μg/mL respectively.

### Antioxidant results of the plant extracts

The results of antioxidant assays of extracts of *N. thomsonianum* using DPPH, ABTS and H_2_O_2_ free radicals are tabulated in Table 2. Overall, the plant extracts observed to possess potent antioxidant properties specifically in DPPH assay.

**Table 2:** Results of antioxidant assays on various crude extracts of *Notholirion thomsonianum*

Samples	Conc (μg/mL)	DPPH Assay		ABTS Assay		H_2_O_2_ Assay	
		% Scavenging (mean ± SEM)	IC_**50**_ (μg/mL)	% Scavenging (mean ± SEM)	IC_**50**_ (μg/mL)	% Scavenging (mean ± SEM)	IC_**50**_ (μg/mL)
Nt.Cr	1000 500 250 125 62.5	80.10 ± 0.95*** 78.70 ± 0.51*** 70.93 ± 0.90*** 60.43 ± 0.70*** 58.26 ± 0.77***	21	71.33 ± 0.88*** 64.66 ± 0.33*** 61.00 ± 0.57*** 56.53 ± 0.40*** 52.46 ± 0.49***	57	64.86 ± 0.85*** 57.63 ± 0.60*** 51.96 ± 0.21*** 45.53 ± 0.61*** 40.63 ± 0.60***	221
Nt.Hex	1000 500 250 125 62.5	83.13 ± 0.80*** 78.83 ± 0.73*** 72.70 ± 0.51*** 66.43 ± 0.70*** 61.06 ± 0.70***	15	53.66 ± 0.88*** 43.66 ± 0.66*** 37.56 ± 0.46*** 32.46 ± 0.49*** 28.66 ± 0.66***	368	54.76 ± 0.78*** 43.16 ± 0.86*** 41.86 ± 0.91*** 37.53 ± 1.09*** 33.16 ± 0.86***	788
Nt.Chf	1000 500 250 125 62.5	88.43 ± 1.26* 83.83 ± 0.66*** 77.93 ± 0.90** 72.26 ± 0.77** 67.10 ± 0.95***	10	74.70 ± 0.55*** 66.20 ± 0.95*** 62.00 ± 0.57*** 55.93 ± 0.44*** 48.53 ± 0.40***	72	69.00 ± 0.57*** 64.33 ± 0.88*** 55.06 ± 1.04*** 52.00 ± 0.57*** 48.53 ± 0.61***	79
Nt.EtAc	1000 500 250 125 62.5	89.96 ± 0.55 ^ns^ 85.93 ± 0.30 ^ns^ 81.40 ± 0.65 ^ns^ 77.46 ± 0.50 ^ns^ 72.10 ± 0.65 ^ns^	< 0.1	67.33 ± 0.33*** 65.50 ± 0.43*** 61.46 ± 0.57*** 53.56 ± 0.60*** 46.20 ± 0.95***	89	76.43 ± 0.81* 73.56 ± 1.24^ns^ 68.46 ± 0.50* 63.60 ± 0.41* 58.33 ± 0.88*	16
Nt.Aq	1000 500 250 125 62.5	68.63 ± 0.70*** 63.06 ± 0.60*** 57.60 ± 0.87*** 52.93 ± 0.50*** 46.76 ± 1.00***	85	51.56 ± 0.49*** 47.40 ± 0.52*** 41.66 ± 0.61*** 38.46 ± 0.54*** 35.56 ± 0.60***	942	43.00 ± 0.57*** 41.40 ± 0.64*** 33.76 ± 0.52*** 29.43 ± 0.59*** 24.76 ± 0.52***	1403
AA	1000 500 250 125 62.5	92.55 ± 0.35 87.84 ± 0.26 81.33 ± 0.88 76.54 ± 0.54 72.67 ± 0.19	< 0.1	87.60 ± 0.40 86.53 ± 0.60 80.43 ± 0.55 78.46 ± 0.58 75.46 ± 0.57	< 0.1	83.33 ± 0.88 74.66 ± 0.49 71.50 ± 0.60 68.00 ± 0.57 65.06 ± 1.04	9.87

In DPPH assay of *N. thomsonianum,* Nt.EtAc exhibited highest activity causing 89.96 ± 0.55, 85.93 ± 0.30, 81.40 ± 0.65, 77.46 ± 0.50 and 72.10 ± 0.65% inhibitions at concentrations of 1000, 500, 250, 125 and 62.5 μg/mL respectively gaining IC_50_ < 0.1 μg/mL. Nt. Chf was comparatively active with 88.43 ± 1.26, 83.83 ± 0.66, 77.93 ± 0.90, 72.26 ± 0.77 and 67.10 ± 0.95% inhibitions at concentrations of 1000, 500, 250, 125 and 62.5 μg/mL respectively, IC_50_ 10 μg/mL. Comparatively, standard drug ascorbic acid showed 92.55 ± 0.35, 87.84 ± 0.26, 81.33 ± 0.88, 76.54 ± 0.54 and 72.67 ± 0.19% inhibitions at the same concentrations gaining IC_50_ of < 0.10 μg/mL.

In ABTS free radicals scavenging assay, Nt. Chf exhibited highest activity of 74.70 ± 0.55, 66.20 ± 0.95, 62.00 ± 0.57, 55.93 ± 0.44, and 48.53 ± 0.40% inhibitions at concentrations of 1000, 500, 250, 125 and 62.50 μg/mL respectively attaining IC_50_ of 72 μg/mL. In this assay, the ethyl acetate fraction (Nt.EtAc) was observed to be less potent than chloroform fraction giving IC_50_ value of 89 μg/mL. The standard drug IC_50_ against the ABTS free radicals was also < 0.1 μg/mL.

Likewise majority of our observations, the Nt.EtAc was also dominant in scavenging the free radicals from H_2_O_2_ as shown in Table 2. Nt. EtAc showed highest activity i.e. 76.43 ± 0.81, 73.56 ± 1.24, 68.46 ± 0.50, 63.60 ± 0.41 and 58.33 ± 0.88% inhibitions at concentrations of 1000, 500, 250, 125 and 62.50 μg/mL respectively. Nt. Chf demonstrated 69.00 ± 0.57, 64.33 ± 0.88, 55.06 ± 1.04, 52.00 ± 0.57 and 48.53 ± 0.61% inhibitory activity at concentrations of 1000, 500, 250, 125 and 62.50 μg/mL respectively. Furthermore, Nt. Cr, Nt. Hex and Nt. Aq exhibited 64.86 ± 0.85, 54.76 ± 0.78 and 43.00 ± 0.57% inhibitions at highest tested concentration. The IC_50_ values of crude extracts were 16 (Nt.EtAc), 79 (Nt.Chf), 221 (Nt.Cr), 788 (Nt.Hex) and 1403 (Nt.Aq) μg/mL. the observed and calculated IC_50_ of standard ascorbic acid was 9.87 μg/mL.

### *α***-Glucosidase inhibitions of isolated compounds**

The isolated compounds (Nt01 – Nt03) from *N. thomsonianum* were evaluated for their in-vitro inhibitory potentials against the *α*-glucosidase enzyme as shown in Table 3. The half maximal inhibitory concentrations (IC_50_) are provided in μg/mL as well as in μM (micromole) based on the molecular weight of each tested compound. Among the isolated compounds, Nt03 showed highest activity with IC_50_ value of 19.54 μM in comparison to the standard drug acarbose (IC_50_ of 10.60 μM). The calculated IC_50_ values of compounds Nt01 and Nt02 were 58.93 and 114.93 μM respectively.

**Table 3:** Results of *α*-glucosidase, *α*-amylase and PTP1B inhibitions of isolated compounds from *w*

Compound	Mol wt	Conc (μg/mL)	***α***-Glucosidase results			***α***-Amylase results			PTP1B results		
			Percent Inhibition (mean ± SEM)	IC_**50**_ (μg/mL)	IC_**50**_ (μM)	Percent Inhibition (mean ± SEM)	IC_**50**_ (μg/mL)	IC_**50**_ (μM)	Percent Inhibition (mean ± SEM)	IC_**50**_ (μg/mL)	IC_**50**_ (μM)
**Nt01**	216	500 250 125 62.50 31.25	55.56 ± 1.06^***^ 51.90 ± 0.45^***^ 44.40 ± 0.82^***^ 31.33 ± 0.66^*^ 21.42 ± 0.43^ns^	272.82	58.93	77.85 ± 2.24^***^ 72.08 ± 0.47^***^ 67.90 ± 0.96^***^ 63.28 ± 0.57^***^ 57.47 ± 0.56^***^	12.15	56.25	90.09 ± 0.32^***^ 88.67 ± 1.20^***^ 83.40 ± 0.25^***^ 78.58 ± 1.12^***^ 74.65 ± 1.34^***^	2.80	12.96
**Nt02**	112	500 250 125 62.50 31.25	76.85 ± 2.24^***^ 71.08 ± 0.47^***^ 66.90 ± 0.96^***^ 61.35 ± 0.51^***^ 57.40 ± 0.76^***^	12.87	114.93	91.36 ± 0.49^ns^ 85.34 ± 0.55^*^ 78.39 ± 0.49^**^ 72.47 ± 0.52^*^ 67.44 ± 0.55^***^	10.81	96.54	93.03 ± 0.48^*^ 90.90 ± 0.48^*^ 85.79 ± 0.63^**^ 79.67 ± 0.61^***^ 75.69 ± 0.77^***^	4.06	36.22
**Nt03**	430	500 250 125 62.50 31.25	78.35 ± 0.23^***^ 73.36 ± 0.84^***^ 70.62 ± 0.25^***^ 66.16 ± 0.16^***^ 59.67 ± 0.32^***^	8.40	19.54	93.08 ± 1.04^ns^ 86.45 ± 0.90^ns^ 80.58 ± 0.63^ns^ 73.40 ± 0.20^ns^ 67.80 ± 0.90^*^	10.49	24.39	90.83 ± 0.47^***^ 87.23 ± 0.96^***^ 82.29 ± 0.57^***^ 78.33 ± 0.55^***^ 76.03 ± 0.77^***^	1.54	3.57
**Acarbose**	645	500 250 125 62.50 31.25	87.08 ± 0.47 82.40 ± 0.20 77.61 ± 0.43 75.45 ± 0.90 63.89 ± 0.20	6.84	10.60	95.23 ± 0.22 89.45 ± 0.90 83.90 ± 0.60 77.00 ± 0.30 72.90 ± 0.45	8.20	12.71	**–**	**–**	**–**
**Ursolic acid**	**–**	**–**	**–**	**–**	**–**	**–**	**–**	**–**	98.65 ± 1.32 93.56 ± 0.45 91.52 ± 0.66 88.22 ± 1.28 86.42 ± 0.43	1.66	3.63

### *α***-Amylase inhibition of isolated compounds**

Our isolated compounds (Nt01 – Nt03) were also good to moderate in inhibiting the *α*-amylase enzyme as shown in Table 3. All the compounds and standard acarbose were tested at concentrations of 500, 250, 125, 62.50 and 31.25 μg/mL. Among the three isolated compounds, Nt03 was the one with highest inhibition potential. The compound Nt03 exhibited IC_50_ value of 24.39 μM (equivalent to 10.49 μg/mL). In comparison, the IC_50_ value given by acarbose was 8.20 μg/mL.

### Protein tyrosine phosphatase 1B (PTP1B) inhibition assay isolated compounds

Besides the two targets (alpha glucosidase and amylase), we also tested our compounds for protein tyrosine phosphatase 1B as shown in Table 3. Based on the observed IC_50_ values in PTP1B target, we can claim that our compounds, specifically Nt03 is an excellent inhibitor in comparison to the standard ursolic acid. The IC_50_ value given by compound Nt03 was 3.57 μM in comparison to the standard drug (ursolic acid IC_50_ was 3.63 μM). Similarly, the compounds Nt01 and Nt02 exhibited IC_50_ values of 12.96 and 36.22 μM respectively.

### Antioxidant potentials of isolated compounds

We also determined the antioxidant potentials of isolated compounds with DPPH free radical scavenging method (Table 4). The observed percent inhibitions at various concentrations of the isolated compounds showed that our compounds have potential antioxidant properties. At highest tested concentration (500 μg/ml), compounds Nt01, Nt02 and Nt03 gave inhibitory potentials of of 88.35, 86.60 and 90.69% respectively. The IC_50_ value exhibited by the ascorbic acid was 1.78 μM (equivalent to 0.314 μg/mL). In comparison to the standard drug, our compounds exhibited IC_50_ values of 4.70 (Nt01), 41.53 (Nt02) and 2.75 μg/mL (Nt03).

**Table 4:** Antioxidant potentials of isolated compounds from *N. thomsonianum*

Compound	Mol wt	Conc (μg/mL)	Percent Inhibition (mean ± SEM)	IC_50_ (μg/mL)	IC_50_ (μM)
Nt01	216	500 250 125 62.50 31.25	88.35 ± 0.89^*^ 84.36 ± 1.15^*^ 79.62 ± 0.03^**^ 76.16 ± 0.12^***^ 73.67 ± 0.35^***^	2.02	4.70
Nt02	112	500 250 125 62.50 31.25	86.60 ± 0.00^***^ 79.32 ± 0.40^*^ 74.78 ± 0.44^***^ 69.08 ± 0.66^***^ 64.40 ± 0.40^***^	8.97	41.53
Nt03	430	500 250 125 62.50 31.25	90.69 ± 0.14^ns^ 87.14 ± 0.49^ns^ 84.44 ± 0.15^ns^ 82.72 ± 0.11^ns^ 79.85 ± 0.17^*^	0.421	2.75
Ascorbic acid	176	500 250 125 62.50 31.25	91.75 ± 0.42 88.47 ± 0.71 85.20 ± 0.49 83.42 ± 1.55 81.62 ± 0.58	0.314	1.78

### Docking studies

#### Docking studies on homology modelled α-glucosidase

**Fig. 2:**
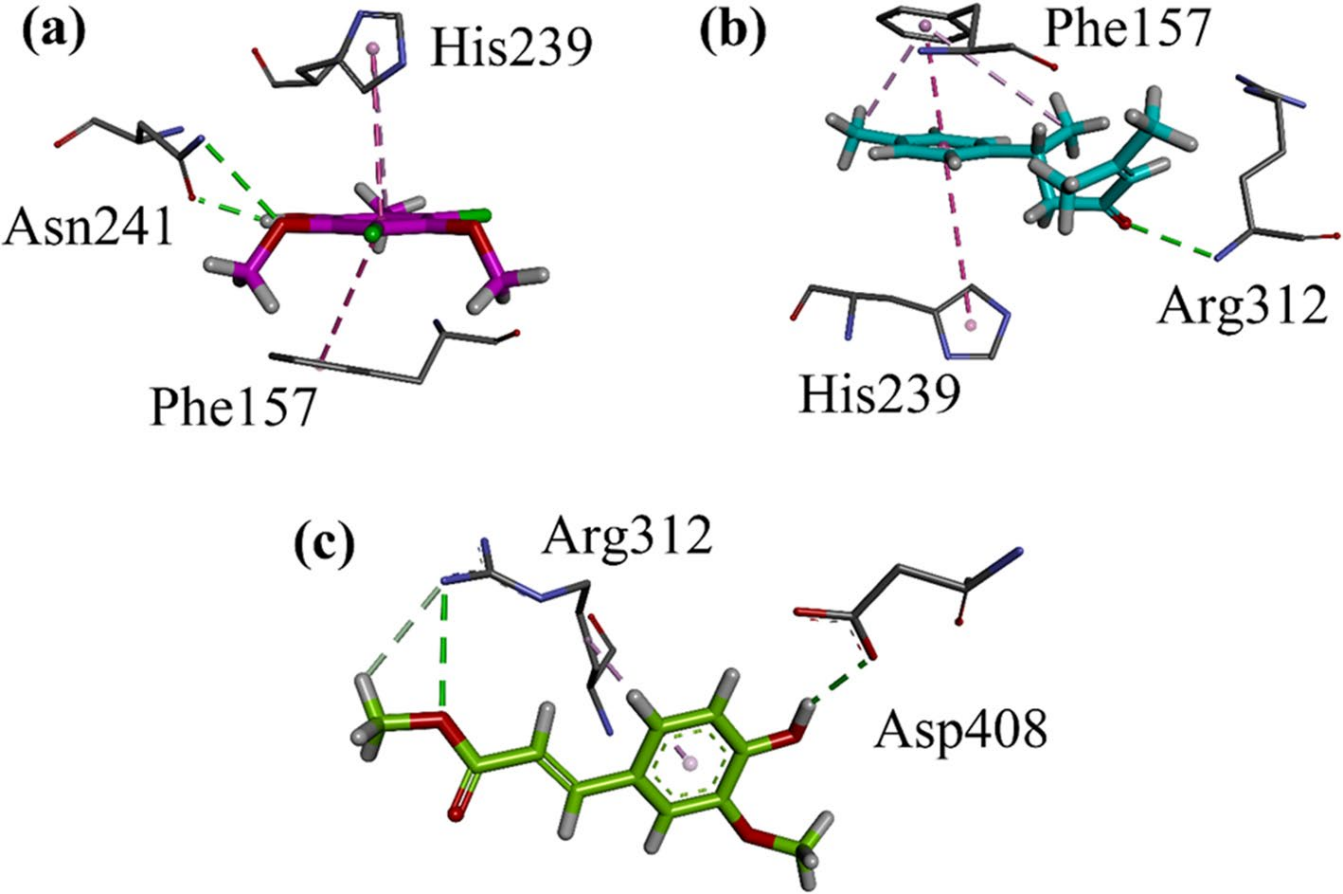
(**a-c**) Close-up 3-D interaction plot of the compounds Nt01-Nt03 into the binding site of homology modelled α-glucosidase

Docking studies on our previously reported homology model was carried out by using Molecular operating Environment (MOE 2016) software package. Three-dimensional (3-D) plots of identified compounds (Nt01- Nt03) into the binding site of homology modelled α-glucosidase are shown in Fig. 2a-c. All the identified compounds show hydrogen bond interactions with important amino acid residues of yeast α-glucosidase model. The important residues involved are Asn241, Arg312, Asp408. The compounds also form π-π stacking interactions. Phe157, His239 are involved in these types of hydrophobic interactions with the phenyl rings of the identified compounds. Moreover, some π-alkyl type of hydrophobic interactions also stabilizes the ligand-enzyme complex. The binding interaction of standard acarbose on homology modelled *α*-glucosidase is shown in Figure S7 of supporting information.

#### **Docking studies on***α***-amylase**

**Fig. 3:**
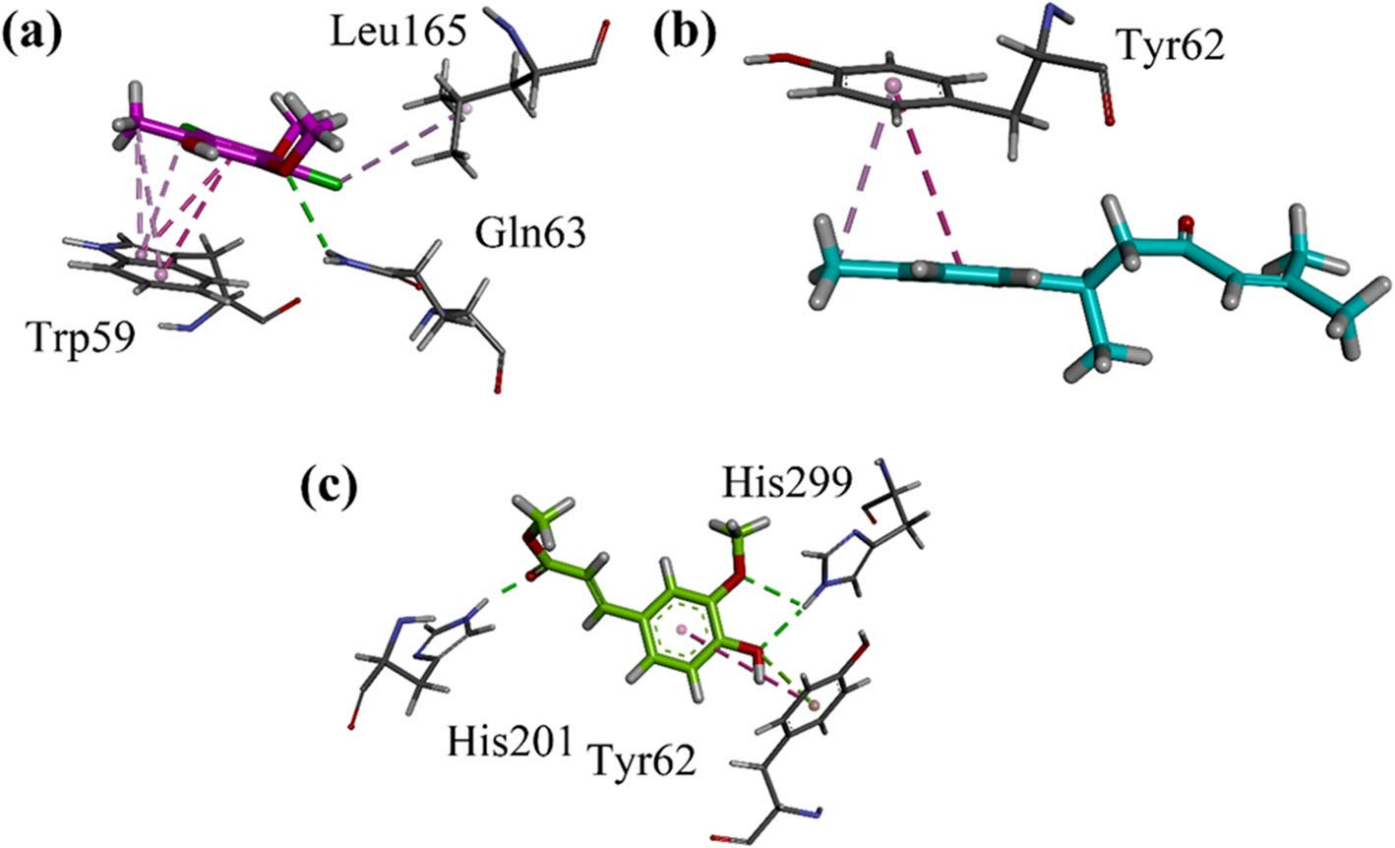
(**a-c**) Close-up 3-D interaction plot of the compounds Nt01-Nt03 into the binding site of human pancreatic α-amylase with accession code 4 W93

Docking studies on Human pancreatic *α*-amylase was performed by using MOE software. Crystal structure of human pancreatic α-amylase with accession code 4 W93 was obtained from PDB [29]. 3-D interaction plots of all the six identified compounds are shown in Fig. 3a-c. The ligand enzyme complexes are stabilized by forming hydrogen bond and π-π stacking interactions. Moreover, hydrophobic π-alkyl type of interactions is also present. Tyr62, a key residue, forms π-π stacking interactions in most cases. Trp59 also forms π-π stacking interactions. While Gln63, His201, and His299 establish hydrogen bond interactions with the studied compounds. The binding interaction of standard acarbose on homology modelled *α*-amylase is shown in Figure S8 of supporting information.

#### Docking studies on protein tyrosine phosphatase 1B (PTP1B)

**Fig. 4:**
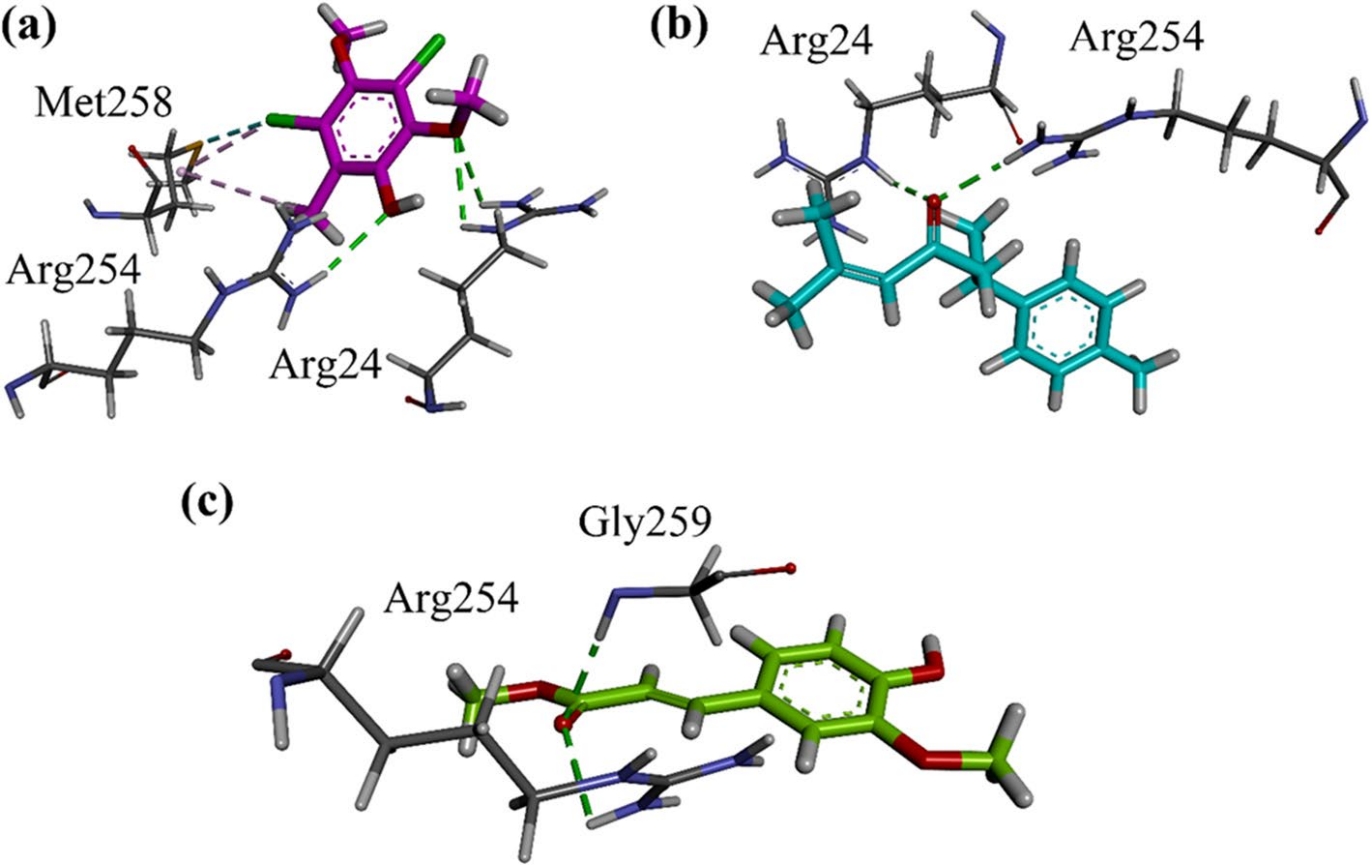
(**a-c**) Close-up 3-D interaction plot of the compounds Nt01-Nt03 into the catalytic binding site of protein tyrosine phosphatase 1B (PTP1B, PDB ID = 1NNY)

Docking studies on Protein tyrosine phosphatase 1B (PTP1B) was performed by using MOE software. PTP1B in complex with catalytic inhibitor was retrieved from protein data Bank (PDB ID = 1NNY). 3-D interaction plots of all the six identified compounds are shown in Fig. 4a-c. Arg24 and Arg254 form hydrogen bond interactions with all the isolated compounds. Gly259 also forms hydrogen bond interaction. Computed binding energies and interacting residues of all the isolated compounds and standard drugs are tabulated in Table 5. The binding interaction of standard ursolic acid on homology modelled PTP1B is shown in Figure S9 of supporting information.

**Table 5:** Binding affinity values and interacting residues of isolated compounds from *N. thomsonianum* toward *α*-glucosidase, *α*-amylase and PTP1B

Compounds	*α*-Glucosidase		*α*-Amylase		PTP1B	
	BE	Interacting residues	BE	Interacting residues	BE	Interacting residues
Nt01	−6.68	Phe157, His239, Asn241	−5.26	Trp59, Gln63, Leu165	−4.93	Arg24, Arg254, Met258
Nt02	−4.09	Phe157, His239, Arg312	−4.52	Tyr62	−3.84	Arg24, Arg254,
Nt03	−7.16	Arg312, Asp408	−5.94	Tyr62, His201, His299	−5.19	Arg254, Gly259
Acarbose	−9.27	Asp214, His279, Glu304, Pro309, Arg312, Asp349, Arg439	−9.57	Trp59, Gln63, Arg195, Asp197, Lys200, His201, Glu233, Glu240, Asp300, Gly306	–	–
Ursolic acid	–	–	–	–	−5.72	Arg24, Arg254, Gly259

*BE* binding energies

## Discussion

Natural products are still believed as a potential source for the discovery of new drugs and play a vital role in the drug development programs [30–32]. Numerous medicinal herbs are rich sources of bioactive compounds that are comparatively free from undesirable side effects and exhibit useful pharmacological actions [32]. Medicinal plants are rich sources of bioactive compounds for the management of various diseases [32–34]. Modern research revealed that more than 411 compounds belonging to different groups including alkaloids, flavonoids, terpenes, phenols, quinines, phenylpropanoids, steroids isolated from medicinal plants, confirm potent inhibitory activity towards α-glucosidase enzymes [35]. With the alarming increase in diabetic population globally, there is a dire need to explore relatively safe and multitarget antidiabetic drugs.

Diabetes mellitus (DM) is a common metabolic disorder, characterized by an abnormal postprandial raise in blood glucose level. Control of postprandial hyperglycemia is believed as an important tool in the management of DM. Parallelly, the antioxidant-based drug therapies are commonly used for the mitigation and treatment of multiple diseases including atherosclerosis, stroke, cancer, Alzheimer’s disease and diabetes [36]. In living systems, free radicals are generated during normal metabolic processes, in mitochondria, during xanthene oxidase activity, liver mixed function oxidases, atmospheric pollutants, from the transitional metal catalysts, xenobiotics and drugs [37]. Additionally, chemical mobilization of the fat stores under diverse circumstances like lactation, infections, fever, fasting and exercise can augment the free radicals generation and activity. Oxidative injury mediated by free radicals is now considered as the fundamental mechanism, causing a large number of the human neurologic and other disorders. Peroxidation of lipids may be initiated by the oxygen free radical, which sequentially stimulates the glycation of proteins, inactivation of some key enzymes and play a vital role in chronic complication of diabetes [18]. So, based on the literature, we initially find out the antidiabetic potentials of crude extracts of *N. thomsonianum* using *α*-glucosidase and *α*-amylase targets. We also supplemented the activities with antioxidant potentials of the crude extracts using free radicals’ methods of DPPH, ABTS and H_2_O_2_. Based on the initial in-vitro screenings of the methanolic extract and subfractions, we observed the ethyl acetate fraction as the potent one. We subjected this fraction to isolation of bioactive compounds and were able to purify three compounds (Nt01 – Nt03). The antidiabetic potentials of the isolated compounds were tested using in-vitro targets of *α*-glucosidase, *α*-amylase, protein tyrosine phosphatase 1B and DPPH. Based on our result, we can claim that our isolated compounds, specifically Nt03 can be lead multitarget antidiabetic agent.

The molecular docking is a powerful dry lab approach to find out the interactions and binding energies of a drug molecule in a drug target [29]. This in-silico approach, in combination with in-vitro analysis, provides an effective base for testing a drug in experimental animals [38]. Herein, we also docked the three isolated compounds against the tested in-vitro targets of *α*-glucosidase, *α*-amylase, and protein tyrosine phosphatase 1B. The molecular docking studies revealed encouraging bind interactions with the target proteins for our compounds.

## Conclusions

It is concluded from our results, that we have explored *N. thomsonianum* as a potential plant for the management of diabetic through multitarget approach. In the initial in-vitro screening of the crude samples from the plant, we observed that ethyl acetate fraction is overall the potent in inhibiting all the tested targets. The bio-guided based approach for the isolation of compounds enables us to purify three compounds (Nt01 – Nt03). We also tested the isolated compounds *α*-glucosidase, *α*-amylase, protein tyrosine phosphatase 1B and DPPH in-vitro targets. Moreover, we also find out the binding interactions with the target proteins using molecular docking approach. The in-vitro and encouraging binding in-silico results revealed that *N. thomsonianum* is a potential source of bioactive compounds as emerging multitarget antidiabetic drugs.

## Supplementary Information

**Additional file 1.**

## Data Availability

The data is available on request from corresponding authors.

## Abbreviations

DM: Diabetes mellitus
PTP1B: Protein tyrosine phophatase 1B
Nt.Cr: *Notholirion thomsoniam* crude extract
Nt.Hex: *n-Hexane* fraction *Notholirion thomsoniam*
Nt.Chf: Chloroform fraction of *Notholirion thomsoniam*
Nt.EtAc: Ethyl acetate fraction of *Notholirion thomsoniam*
Nt.Aq: Aqueous fraction *Notholirion thomsoniam*
DPPH: 2,2-diphenyl-1-picrylhydrazyl
ABTS: 2,2′-azino-bis(3-ethylbenzothiazoline-6-sulfonic acid)
H_2_O_2_: Hydrogen peroxide
AGIs: Alpha glucosidase inhibitors
ROS: Reactive Oxygen Species
CVD: Cardiovascular disease
NMR: Nuclear Magnetic Resonance Spectroscopy
MS: Mass spectrometry
*J*: Coupling constant
AA: Ascorbic acid
MOE: Molecular Operating Environment
PDB: Protein Data Bank
