# Supplementary material for: Crude extract and isolated bioactive compounds from Notholirion thomsonianum (Royale) Stapf as multitargets antidiabetic agents: in-vitro and molecular docking approaches
Source: BMC Complement Med Ther. 2021 Oct 27;21:270. doi: 10.1186/s12906-021-03443-7 (PMC8549260; doi:10.1186/s12906-021-03443-7)

**Crude extract and isolated bioactive compounds from *Notholirion* *thomsonianum* (Royale) Stapf as multitargets antidiabetic agents: *In*-*vitro* and molecular docking approaches**

Mater H. Mahnashi^1^ ([matermaha@gmail.com](mailto:matermaha@gmail.com))

Yahya S. Alqahtani^1^ ([yahyasalqahtani0@gmail.com](mailto:yahyasalqahtani0@gmail.com))

Ali O. Alqarni^1^ ([aoqarni@gmail.com](mailto:aoqarni@gmail.com))

Bandar A. Alyami^1^ ([alyamibandar1@gmail.com](mailto:alyamibandar1@gmail.com))

Muhammad Saeed Jan^2^ ([saeedjanpharmacist@gmail.com](mailto:saeedjanpharmacist@gmail.com))

Muhammad Ayaz^3^ ([ayazuop@gmail.com](mailto:zeb.takor@yahoo.com))

Farhat Ullah^3^ ([farhataziz80@hotmail.com](mailto:zeb.takor@yahoo.com))

Umer Rashid^4^ ([umerrashid@cuiatd.edu.pk](mailto:umerrashid@cuiatd.edu.pk))

Abdul Sadiq*^3^ ([sadiquom@yahoo.com](mailto:zeb.takor@yahoo.com))

^1^Department of Pharmaceutical Chemistry, College of Pharmacy, Najran University, Najran, Saudi Arabia. ^2^Department of Pharmacy, University of Swabi, KP, Pakistan. ^3^Department of Pharmacy, Faculty of Biological Sciences, University of Malakand, Chakdara, 18000 Dir (L), KP, Pakistan. ^4^Department of Chemistry, COMSATS University Islamabad, Abbottabad Campus, 22060 Abbottabad, Pakistan.

**Corresponding author:** *Dr. Abdul Sadiq, Associate Professor, Department of Pharmacy, University of Malakand, Chakdara, 18000 Dir (L), KP, Pakistan, Contact: +92(0)301 2297 102, Email: [sadiquom@yahoo.com](mailto:sadiquom@yahoo.com)

**Supplementary Information**

**Figure S1:** MS chromatogram of **Nt01** isolated from *Notholirion* *thomsonianum*.

**
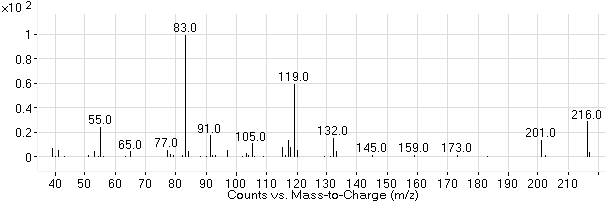
**

**Figure S2:** ^1^H NMR of **Nt01** isolated from *Notholirion* *thomsonianum*.

**
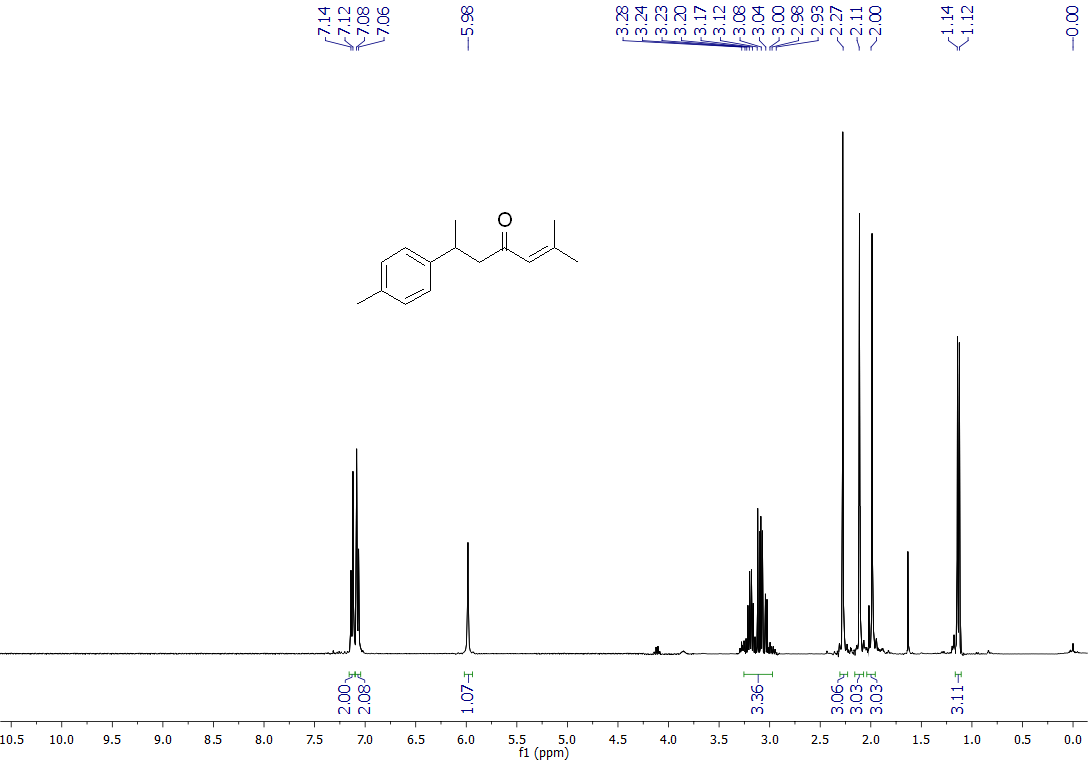
**

**Figure S3:** MS chromatogram of **Nt02** isolated from *Notholirion* *thomsonianum*.

**
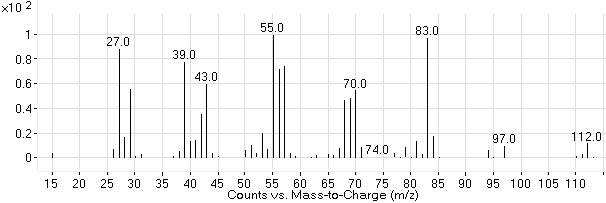
**

**Figure S4:** ^1^H NMR of **Nt02** isolated from *Notholirion* *thomsonianum*.

**
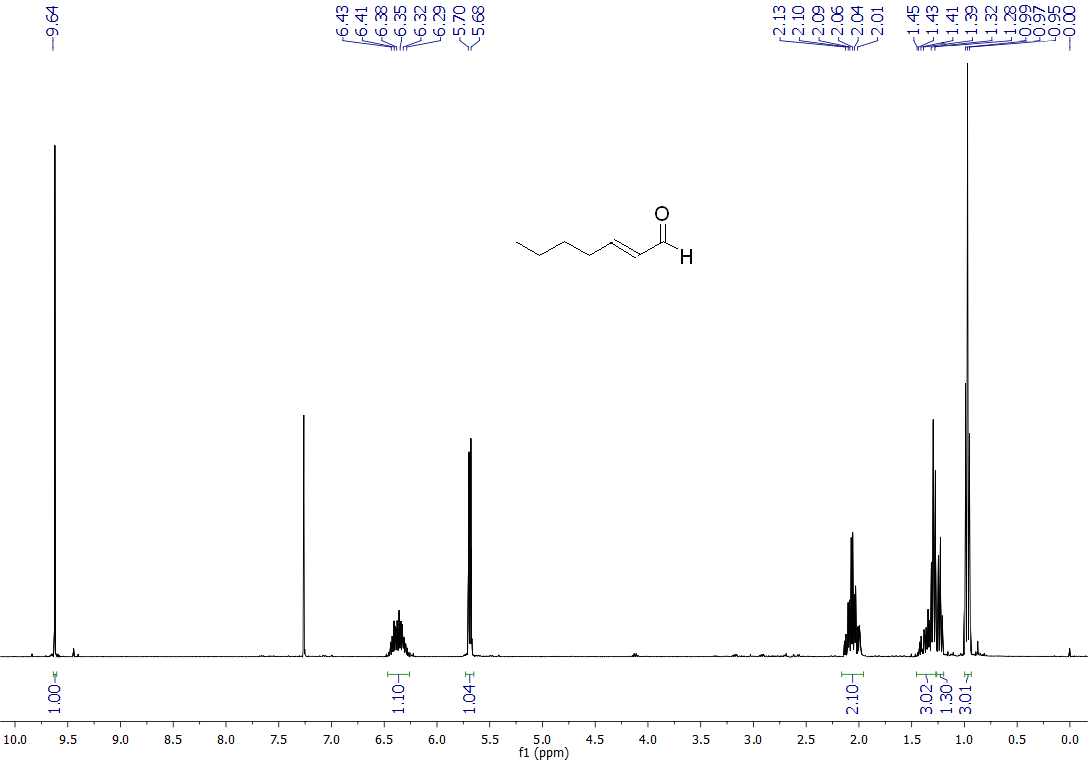
**

**Figure S5:** MS chromatogram of **Nt03** isolated from *Notholirion* *thomsonianum*.

**
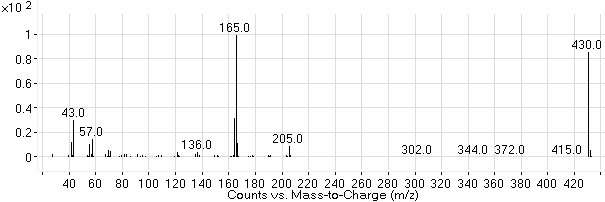
**

**Figure S6:** ^1^H NMR of **Nt03** isolated from *Notholirion* *thomsonianum*.


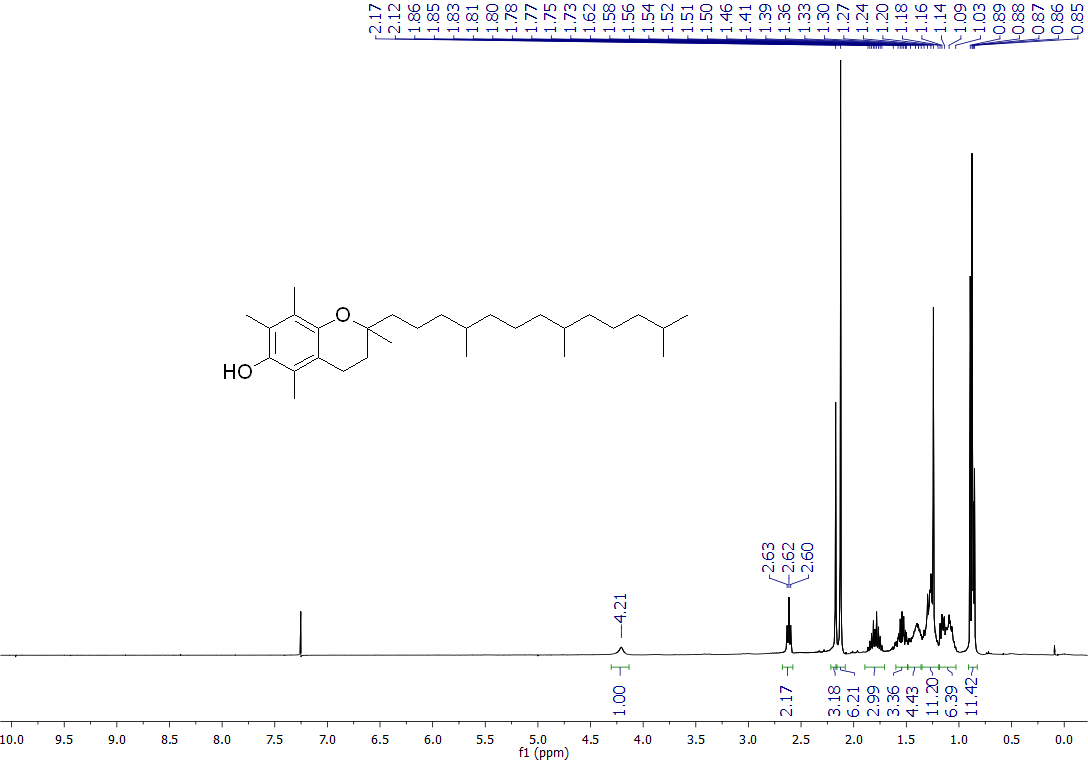


**Figure S7:** Close-up 2-D interaction plot of the compounds **standard drug Acarbose** into the binding site of homology modelled α-glucosidase.


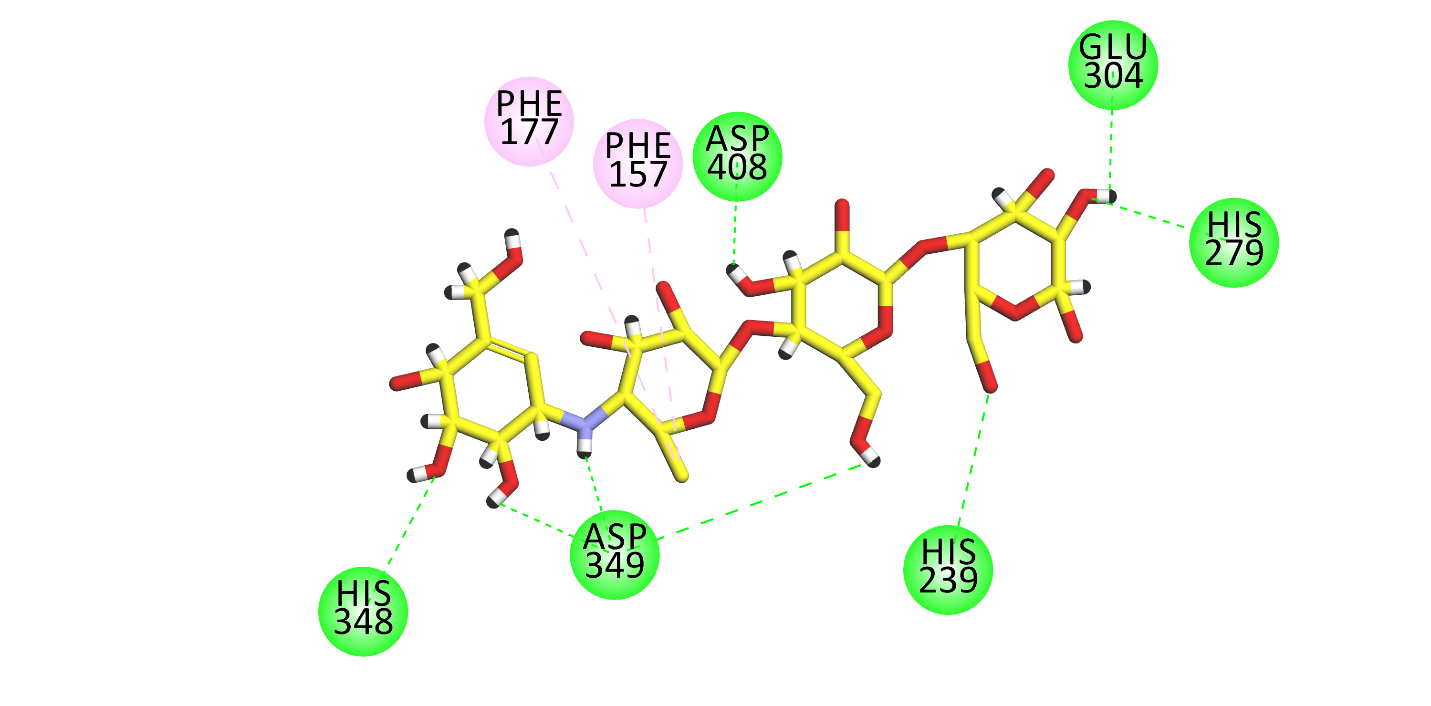


**Figure S8:** Close-up 2-D interaction plot of the compounds **standard drug Acarbose** into the binding site of homology modelled α-amylase.


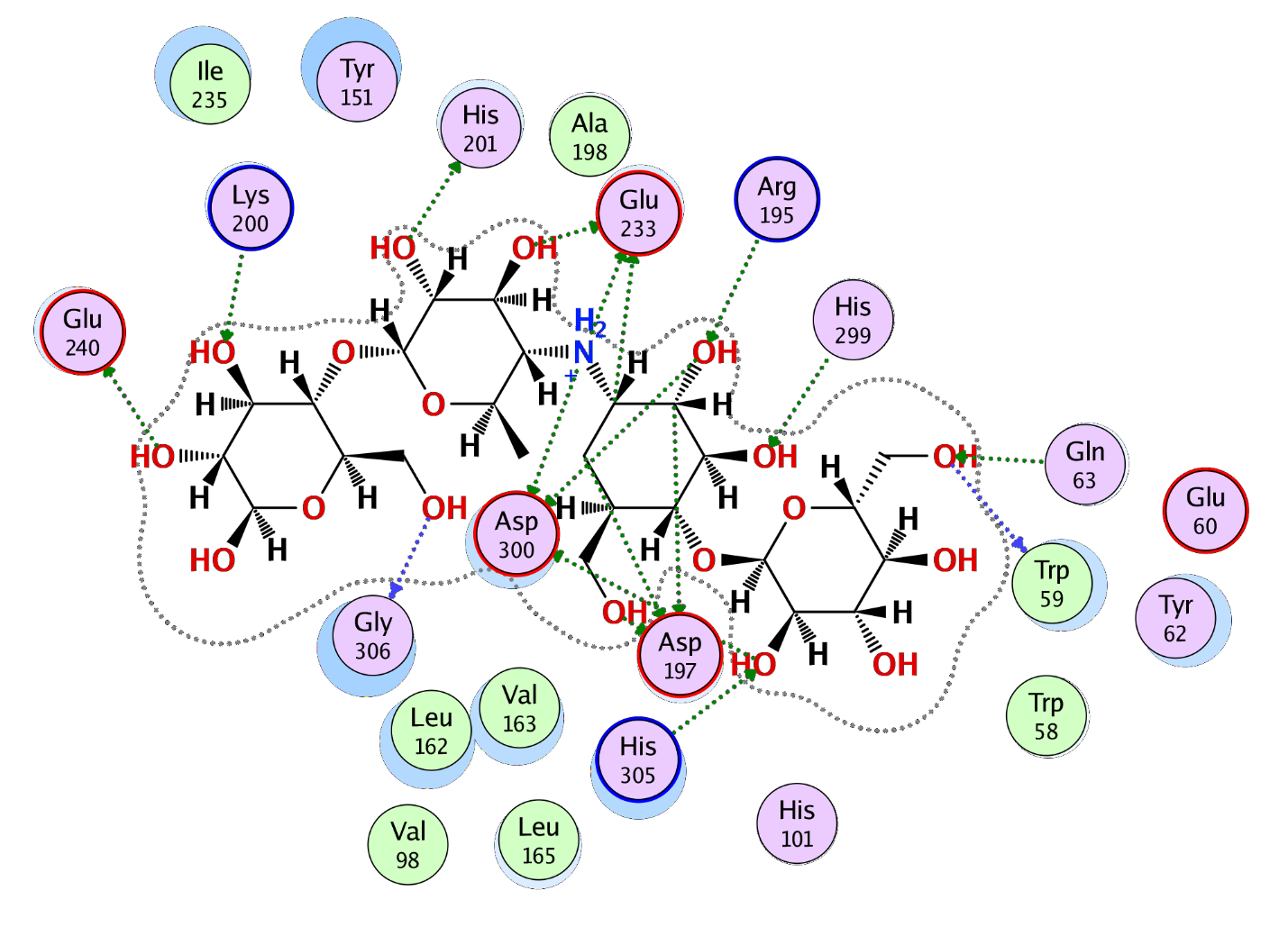


**Figure S9:** Close-up 2-D interaction plot of the compounds **standard drug Ursolic acid** into the binding site of homology modelled PTP-1B.


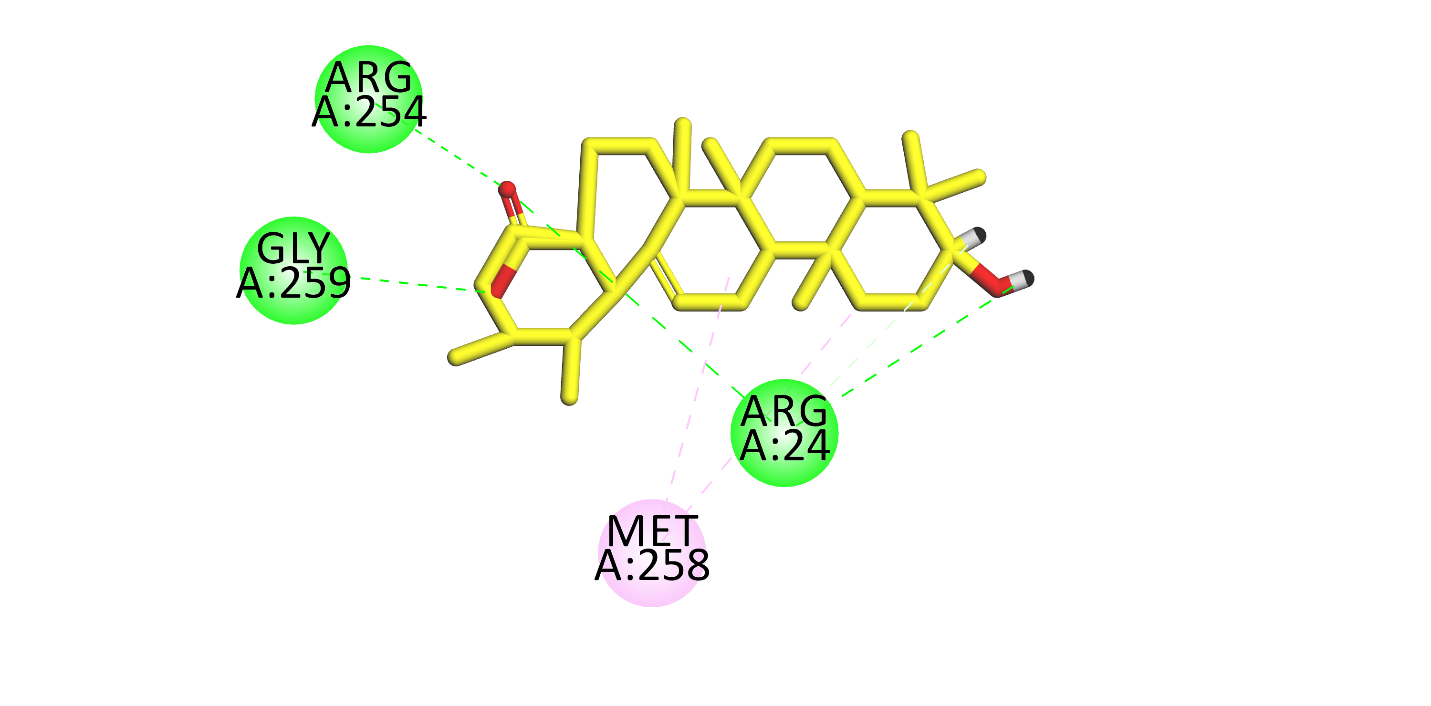

Supplement: Supplementary file 1 — Additional file 1. [file 12906_2021_3443_MOESM1_ESM.docx]
